# Reframing Fish Passage Prioritization for Human Nutrition Outcomes

**DOI:** 10.1007/s00267-025-02271-6

**Published:** 2025-09-13

**Authors:** Nicolette Duncan, Ana Horta, John Conallin, Tim Marsden, Abigail J. Lynch, Ivor Stuart

**Affiliations:** 1 Gulbali Institute, Charles Sturt University, Albury, NSW Australia; 2 Australasian Fish Passage Services Pty Ltd, Newcastle, NSW Australia; 3 U.S. Geological Survey, National Climate Adaptation Science Center, Reston, VA USA

## Abstract

Water control infrastructure forms barriers that fragment river habitats, reducing aquatic biodiversity and the ecosystem services it provides. Irrigation infrastructure, for example, although implemented to support food production, highlights problematic trade-offs against wild food systems like inland fisheries which are a critical food resource for tens of millions of people, particularly in tropical countries. To reduce fragmentation at a broad range of barriers, fish passage technology is sometimes implemented to support migrating fish, aided by frameworks designed to prioritize barriers for remediation. This study critically evaluated 93 fish passage barrier prioritization frameworks globally to explore how they could strategically guide fish passage investments in tropical contexts and identify criteria relevant to delivering on nutrition security outcomes. Results showed prioritization frameworks were ill-equipped to support the broader human development goals that may drive fish passage investments in tropical countries, such as supporting human nutrition under United Nations Sustainable Development Goal (SDG) 2: Zero Hunger. Tropical contexts were underrepresented despite substantial recent fish passage investment, whereas temperate and conservation focused frameworks, particularly from North America, dominated. These findings prompt reflection on the inherent biases in fish passage barrier prioritization frameworks and criteria. Improving understanding of and collaboration with local partners to integrate SDG 2 into future prioritization frameworks could improve fish passage infrastructure and help support better nutrition and food production for communities.

## Introduction

**Fig. 1:**
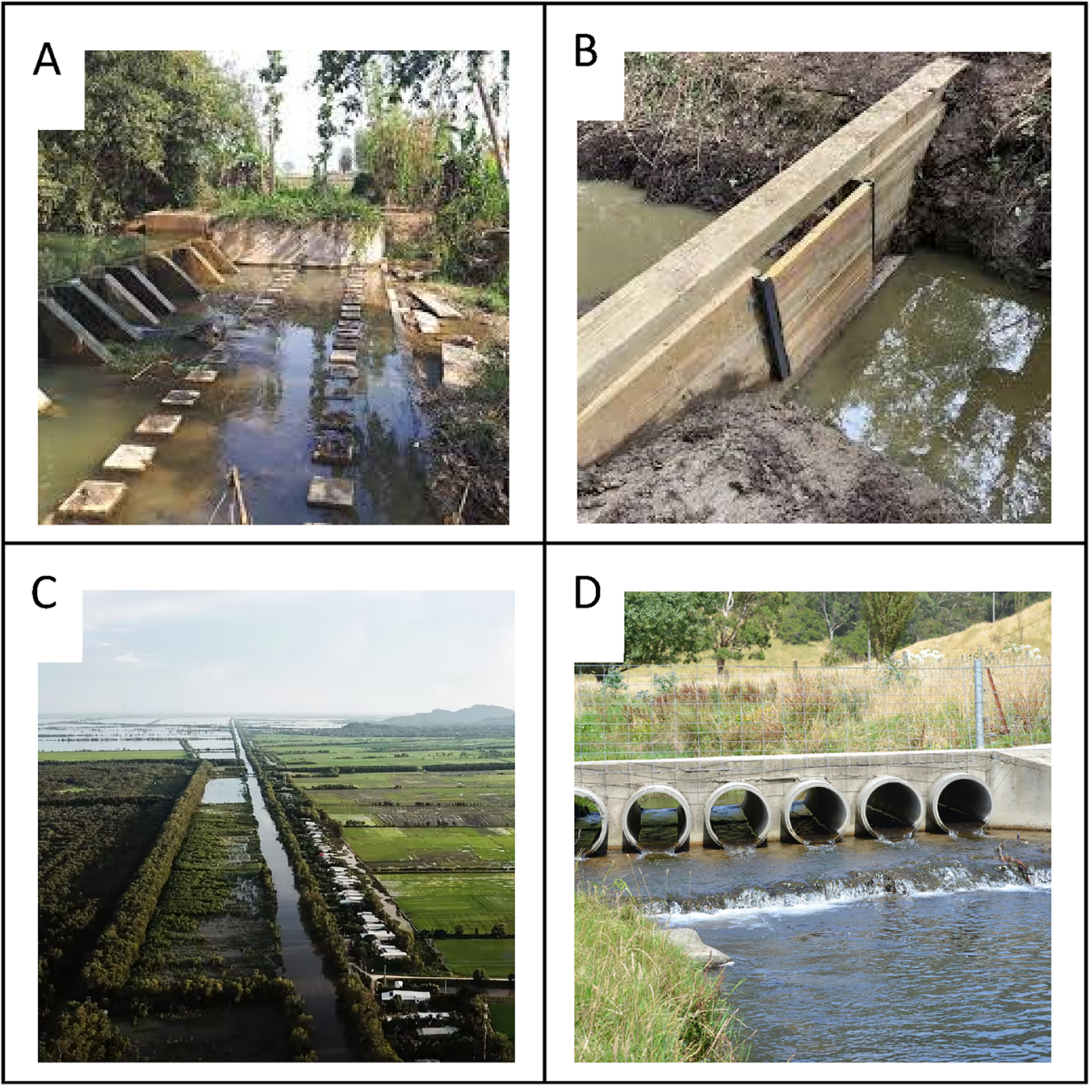
Infrastructure like **A** weirs (Asian Development Bank (ADB) 2020a); **B** sluice gates (Countryside Contracts 2020); **C** canals (Board 2022); and **D** road culverts (author photo) fragment river and floodplain aquatic ecosystems, preventing fish from reaching breeding, spawning, and feeding grounds

Rivers and floodplains have historically benefited and continue to benefit human societies by providing nutritious aquatic foods, water for agriculture, and hydropower generation among many other services (Arthur et al. 2015; Grill et al. 2019). Dams, weirs, and floodplain infrastructure (Fig. 1) substantially affect river systems and the ecosystem services they provide by changing the timing and magnitude of water flows, and fragmenting aquatic habitats (McCartney et al. 2019). Fragmentation prevents or restricts free movement of aquatic organisms including migratory fish to diverse habitats within the river catchment (Fuller et al. 2015). The combined effect of large irrigation infrastructure is substantial, accounting for 70% of freshwater abstraction (McDermid et al. 2023). Simultaneously, underestimation of the effect of hundreds of thousands of small water infrastructure barriers (<10 m in height) indicates abstraction rates may be much higher than previously realized (Baumgartner et al. 2021). Almost 70% of area under irrigation is in Asia, particularly China and India, but also southeast Asia in the Lower Mekong Basin (LMB) where it mainly supports rice agriculture (McDermid et al. 2023).

**Fig. 2:**
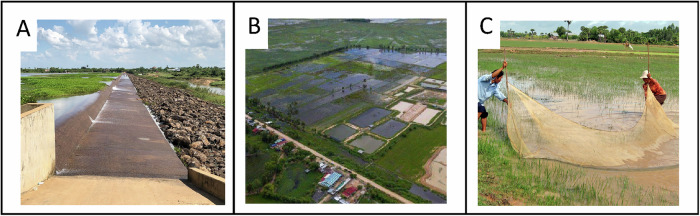
Where water infrastructure prevents fish migration (**A** - UNDP 2024), fish cannot reach floodplain fishing grounds including rice fields (**B** - Bozek 2020), negatively affecting people fishing there (**C** - WorldFish 2017). Where fish significantly contribute to the diets of local people, this can undermine local nutrition security

Water infrastructure development in the LMB is typical of the global dynamics of river regulation. Traditionally, the LMB’s tropical climate and extensive annual flooding drove immense biological productivity and strongly connected local human livelihoods to fisheries and flooded agriculture (Morton and Olson 2018). In recent years, inland fisheries have increasingly faced multiple pressures, with water infrastructure being a major threat (Reid et al. 2019; Stokes et al. 2025). Presently thousands of water infrastructure barriers fragment aquatic habitats (McCartney et al. 2019). Up to 86% of the LMB’s floodplain has been converted to rice agriculture (Fig. 2), with expansion driving reclamation of natural wetlands, loss of floodplain vegetation (Carling 2009), and contributing to fisheries decline (Dudgeon 2005; Halls and Hortle 2021). This decline affects mainly family fishers, especially women and children using the relatively safe and accessible floodplain habitats for subsistence (Garaway et al. 2013; Freed et al. 2020; Nurhasan et al. 2022). While some communities benefit from intensified rice production, others are made more vulnerable through loss of free, accessible, and nutritious wild capture fisheries (Duncan et al. 2021; Friend et al. 2012). The social and ecological trade-offs of irrigation and development are acknowledged in the United Nations (UN) Sustainable Development Goals (SDGs), which emphasize socially inclusive modes of production and development that occur within planetary boundaries to improve present and future people’s wellbeing (United Nations [UN] 2015). Specifically, SDG 2: Zero Hunger, but also SDG 1: No Poverty, SDG 3: Good Health, SDG 5: Gender equality, and SDG 15: Life on land (including freshwater resources) are undermined by the loss of inland capture fisheries as a critical food resource including for vulnerable or marginalized peoples who may not receive full benefit from profit driven development (Bush 2008; Elliott et al. 2022).

SDG 2: Zero Hunger sets the ambitious aim of providing a reliable and adequate supply of nutritious food for all. Nutrition security is a central concern in the LMB’s least developed countries, Cambodia and Lao People’s Democratic Republic (PDR), where iron, vitamin A, calcium, omega 3 fatty acid, and zinc deficiencies are problematic (Barrett 2020; Nurhasan et al. 2010; UNICEF 2021). Nutrition security refers to the provision of essential dietary nutrients, as opposed to food security which relates to providing adequate daily caloric (energy) intake, but not the nutritional quality of food (Simelane and Worth 2020). Nutrition security is multidimensional, including nutrient supply, but also access, utilization, and production sustainability (Simelane and Worth 2020). Globally, reliable access to adequate nutritious food is a major challenge (FAO et al. 2022), and capture fisheries have traditionally filled an important role as abundant, accessible, sustainable, and socially acceptable food (Arthur et al. 2015; Youn et al. 2014). Many fish contain nutrients often lacking in disadvantaged people’s diets (Funge-Smith and Bennett 2019), including all those essential for women’s and children’s health in the first 1000 days of human life (Nurhasan et al. 2022). Wild fish are more nutritious than aquaculture fish, owing to species diversity and nutrient density in small fish which are rarely cultured and are viewed as having low commercial value, but which constitute the majority portion of capture fisheries yield in the LMB (Belton and Thilsted 2014; Nam et al. 2009). The superior nutrition of wild fish underlines the importance of inland fisheries to the nutrition security of marginalized groups until more accessible, sustainable, and equitable alternative food systems can be developed (Tezzo et al. 2018). It is paradoxical that the supply of nutritious fish is potentially being sacrificed for nutrient-poor irrigated rice, with the required water infrastructure often justified based on its production of rice, but failing to account for the loss of fisheries. This is especially problematic where people may have restricted access to alternative nutritious food, either economically or because of their location (Duncan et al. 2021; Friend et al. 2012). Rather, both fish and rice can be maintained in the landscape with appropriate management (McCartney et al. 2019; Freed et al. 2020). Consequently, investments in fishery restoration are taking place to maintain migratory fisheries and protect the nutrition security they provide (ACIAR 2024; ADB 2020b). Fishery restoration interventions target ecological, social, and technical aspects of fisheries, such as habitat restoration, community management, and fish-friendly infrastructure (Mekonen et al. 2025; Rogosch et al. 2024). Their influence on food security varies and is often shaped by local socio-political contexts (Nguyen-Khoa et al. 2020; Arthur et al. 2023). For instance, while aquaculture can boost fish supply, it often has reduced nutritional output and requires land and capital investments that limit broader benefits (Belton and Thilsted 2014; Tezzo et al. 2018). In contrast, fish passage technologies restore connectivity in regulated rivers and can support the >70% of LMB fish species that migrate (Conallin et al. 2019). These technologies benefit shared fisheries resources, including for marginalized groups with limited land or capital (Basurto et al. 2025; Hall et al. 2013; Thilsted et al. 2016). Fish passage can also amplify the effectiveness of other interventions, such as floodplain restoration, by enabling fish to reach breeding and nursery habitats. Without connectivity or habitat quality, ecological gains can be lost (Piczak et al. 2023). Similarly, if fishing is unregulated, fish populations may decline despite improved access to quality habitat. Thus, fish passage is a foundational intervention that supports other restoration efforts. This paper focuses on how fishways are implemented to support both fish populations and human communities.

Given the extent of fragmenting barriers, which are estimated to number ~13,000 in the Mekong basin alone (Sun et al. 2024), and limited financial, technical, and human resources with which to remediate them, fish passage barrier prioritization frameworks have been developed to guide strategic fishway implementation. Prioritization frameworks enable systematic consideration of barriers within river basins in relation to their physical characteristics and environmental context (Kemp and O’Hanley 2010). They do so via a hierarchical process using one or a combination of methods (e.g., rank and score, geographic information systems [GIS], optimization modeling; refer to Table 1) and aim to aid decision-makers in choosing the most important and best value barriers to remediate according to local management objectives. *Rank and score* methods evaluate barriers according to programmed criteria, for example, budget constraints, maximizing connected habitat area, or prioritizing barriers of certain heights (Kemp and O’Hanley 2010). The simple format and reporting of this approach are advantageous however, rank and score approaches neglect relationships between barriers within catchments (Flitcroft et al. 2019; Virbickas and Kesminas 2024). Ignoring the catchment context, specifically the spatial relationship between barriers is problematic given fish passage is about restoring strongly relational habitat connectivity and processes (e.g., Stanford and Ward 1992). In contrast, *Geographic Information Systems (GIS)* methods analyze the spatial relationships between barriers and landscape features in catchments (Cote et al. 2009); however these are constrained by available spatial data which can be limited in developing country contexts (Ali et al. 2025), for example in Cambodia. Optimization approaches use statistical modeling to determine priority barriers in the river network but typically require expert programming and operation which can limit their accessibility to diverse users (Kemp and O’Hanley 2010). Some *hybrid* frameworks combine spatial information in rank and score (e.g., upstream habitat length and barrier characteristics – Atkinson et al. 2020) or in optimization modelling with other habitat, hydrologic, and/or socio-economic data (e.g., cost of fishway construction) which can enhance their applicability in data poor scenarios by drawing from a broader database (e.g., Oregon Department of Fish and Wildlife 2019; Marsden et al. 2023).

**Table 1:** Strengths and limitations of the main methods used to prioritize fish passage barriers for remediation

Prioritization approach	Strengths	Limitations	Examples
Rank and score	• Simple process and reporting	• Considers barriers individually	Catchment Solutions (2021) Copper River Watershed Project (2011) Lin et al. (2020) McKay et al. (2013) O’Brien et al. (2006) Virbickas and Kesminas (2024)
Geographic Information Systems (GIS)	• Considers relationships between features in a catchment	• Limited by spatial data availability	Baumgartner et al. (2021) Carter et al. (2007) Lawson et al. (2010) Martin (2019) Segurado et al. (2015)
Optimization	• Can integrate diverse data inputs	• Requires expert design/operation • Limited by inputs chosen	Barry et al. (2018) Buddendorf et al. (2019) Fitzpatrick and Neeson (2018) Ioannidou and O’Hanley (2019) Perkin et al. (2020) U.S. Fish and Wildlife Service (2016)
Hybrid	• Benefits from diverse data inputs • Useful in poor data settings	• Limited by inputs chosen	Hoenke et al. (2014) Mader and Maier (2008) Maitland et al. (2016) Marsden et al. (2023) Oregon Department of Fish and Wildlife (2019) Ziv et al. (2012)

Prioritization frameworks can be designed by resource management agencies, (e.g., U.S. Fish and Wildlife Service 2016), environmental consultants (e.g., Catchment Solutions), fishway implementers (e.g., Marsden et al. 2023), or academic researchers (e.g., Ioannidou and O’Hanley 2019) to support natural resource managers or funding organizations making fish passage decisions. Some are published in peer-reviewed journals while others are produced by natural resource management agencies as internal documents to standardize barrier prioritization in river basins under their remit. Many frameworks exist, some dating back several decades (Paulsen and Wernstedt 1995; Wernstedt and Paulsen 1995); however, evaluations of their effectiveness are challenging to find (the authors did not locate any although restoration action effectiveness is examined in Rogosch et al. (2024) and fish community recovery like changes in other ecological processes can take years to be realized (Rosenfeld and Hatfield 2006). Over time, methods to identify barriers have improved, and have been supplemented with basic fish surveys to characterize local fisheries to support prioritization. Most prioritization frameworks focus on North American salmonid fisheries (e.g., O’Hanley et al. 2013; US Fish and Wildlife Service 2016; Atkinson et al. 2020) where billions of dollars have been spent to conserve fish stocks threatened by water infrastructure development (Denkmann and Leibovitz 2024). Likewise, abundant research into salmonids, a temperate climate species, sharply contrasts with lack of investment demonstrated in megadiverse tropical regions like the LMB where much less data exist but people are highly dependent on fish for food (Cowx et al. 2025).

Data and metrics used to inform barrier prioritization can privilege some communities over others (King and Fonner 2024). Commonly, barrier prioritization frameworks use upstream habitat length as a decision-making metric, sometimes supported by other technical (e.g., presence of a downstream barrier) or ecological metrics (e.g., surrounding land use, flow metrics). Alone, these metrics lend themselves to maximizing biological processes in rivers, and benefiting river fishers, however social (e.g., nutritional, economic, and traditional) outcomes at best only indirectly result. The lack of socially, or nutrition sensitive metrics implies families and subsistence fishers, many of whom use off-channel fishing grounds like floodplain rice fields (Freed et al. 2020) commonly overlooked in river focused prioritizations, are unaccounted for. Theoretically, metrics relating to habitats or species fished by SDG focal demographics could be an entry point for integrating nutrition security into prioritization frameworks (Conallin et al. 2019), however it is unclear if this is occurring. Alternatively, social demographic, health or livelihoods data could be leveraged to understand spatial patterns of fish dependency and prioritize barriers that might represent greater community benefits if remediated for fish passage (Table 2). Expansion of fisheries management intervention evaluation to include social metrics is observable in policies of influential partners like the National Oceanic and Atmospheric Administration (NOAA 2021) in their Human Integrated Ecosystem Based Research Strategy and the United Nations Food and Agriculture Organization’s Ecosystem Approach to Fisheries (FAO 2025). Where interventions aim to maintain fish productivity for nutrition security as in the case of fish passage restoration in the Mekong, integration of social data could help inform evaluation as the logical next step of barrier prioritization and impact evaluation.

**Table 2:** A non-exhaustive list of existing examples of metrics that could capture the social importance of fisheries

Metrics	Method
Serves undernourished communities	Integrate human malnutrition data from health authorities or development organizations.
Serves poor communities	Integrate community socio-economic data produced by local governments or development organizations.
Serves fishery dependent communities	Integrate fish landings, market, or other data sources that indicate the importance of fisheries to local communities.
Supports fish production for women and children’s health Diet quality	Integrate demographic and health authority data on women and/or children’s nutrition levels where available. Measuring food production and consumption diversity, or nutrient supply adequacy in relation to dietary need.

Such metrics may contribute to better integration of human nutrition in fish passage barrier prioritization. Adapted from Melesse et al. (2020) and NOAA (2021)

Our goal was to conduct a scoping review to better understand fish passage prioritization framework metrics and to identify criteria relevant to supporting and delivering on nutrition security outcomes generally, and specifically in the LMB. Our findings point to the emergence of nutrition security as a motivating factor for fish passage investment in the LMB, but that relevant metrics are underrepresented in frameworks both developed for the LMB, and globally. The present work has implications relevant to fish migration, fish passage, and international development disciplines beyond current fish passage investments in the LMB region. These metrics will be relevant to future investments in tropical and developing regions, and in a global environmental policy context where natural resource management of fish and rivers increasingly needs to contend with multiple, competing demands of resource beneficiaries (refer to Lynch et al. 2025).

## Methods

**Fig. 3:**
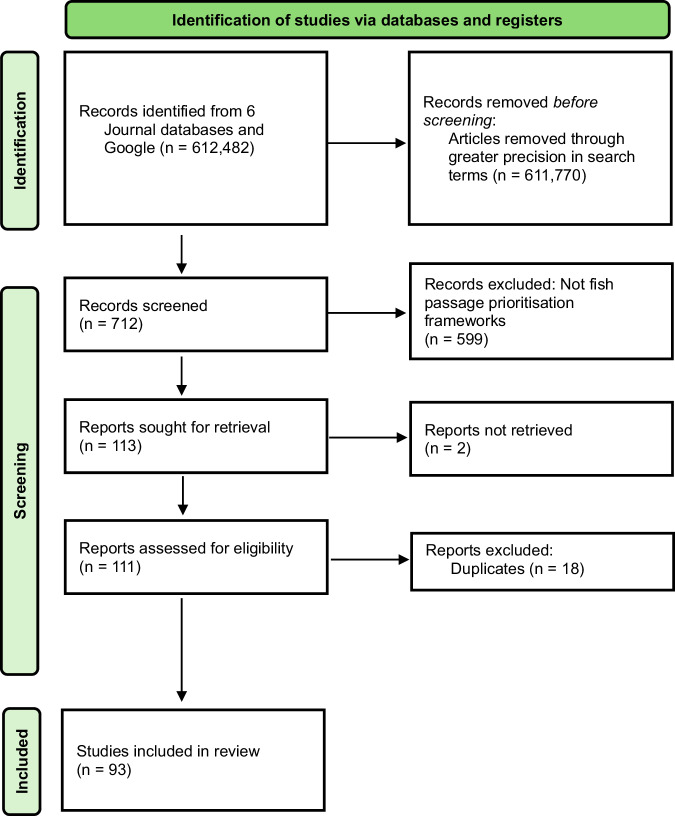
Preferred reporting items for systematic reviews and meta-analyses for scoping review (PRISMA-ScR) flow diagram (Page et al. 2021) of the literature review undertaken to identify publications detailing proposed method steps to identify priority barriers to fish passage (i.e. fish passage barrier prioritization frameworks)

We conducted a scoping review following the Preferred Reporting Items for Systematic Reviews and Meta-Analyses – Scoping review (PRISMA- ScR) guideline (Page et al. 2021). Five journal databases (EBSCO Host, GreenFile, ProQuest, Science Direct, and Taylor and Francis Online), Google Scholar, and Google were manually searched without date restrictions for peer-reviewed articles, and grey literature. A total of 612,482 articles were initially identified (Fig. 3). Of these, 611,770 were excluded before screening by revising search terms in line with our research objectives to increase precision (e.g., “fish passage” AND “prioritization” AND “framework” AND “Mekong”, Google scholar = 2240 items were revised to “fish passage” AND “prioritization framework” AND “Mekong”, Google scholar = 52 items). Both American and British spellings were searched. The remaining 712 articles were screened by title, abstract, and main text to gauge relevance. Of these, 599 records were excluded as not relating to fish passage prioritization (e.g., aquaculture, biology, chemistry, fishway design, medical, social science studies). Two of the remaining 113 records could not be retrieved and 18 were duplicates. Ninety-three records were identified presenting a step-by-step process to support systematic barrier prioritization for improved fish passage and were analyzed (Fig. 3).

**Fig. 4:**
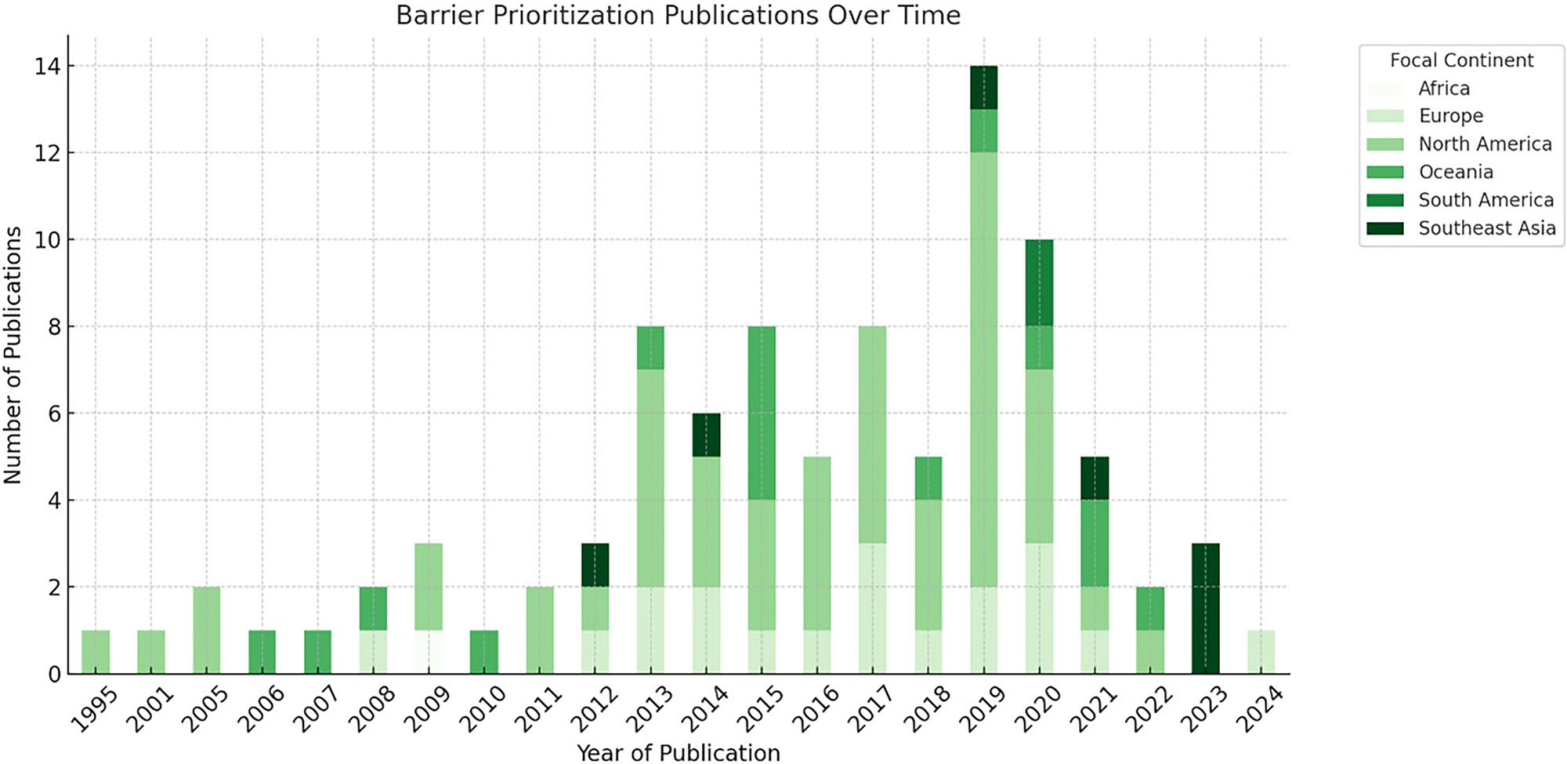
Frequency distribution of barrier prioritization frameworks, identified in the present study, published over time and according to geographic area

**Fig. 5:**
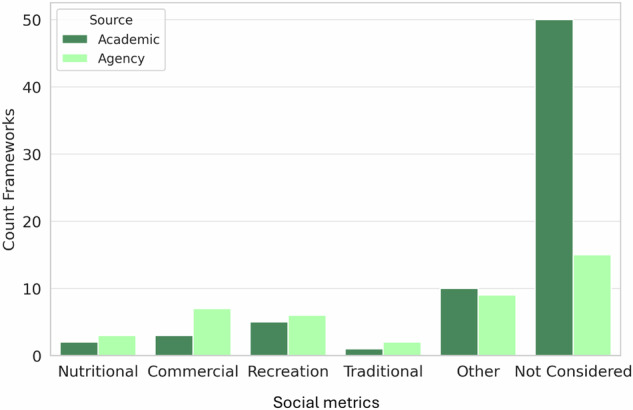
Frequency distribution of the social metric criteria used by reviewed barrier prioritization frameworks reviewed in this study. ‘Other’ values included dam safety (mentioned in 4% of total frameworks), stakeholder support for infrastructure remediation (4%), water/hydropower security (4%), willingness to pay for ecosystem services (1%), livelihoods (1%), as well as less well-defined productivity benefits (3%) and social goals (2%)

**Fig. 6:**
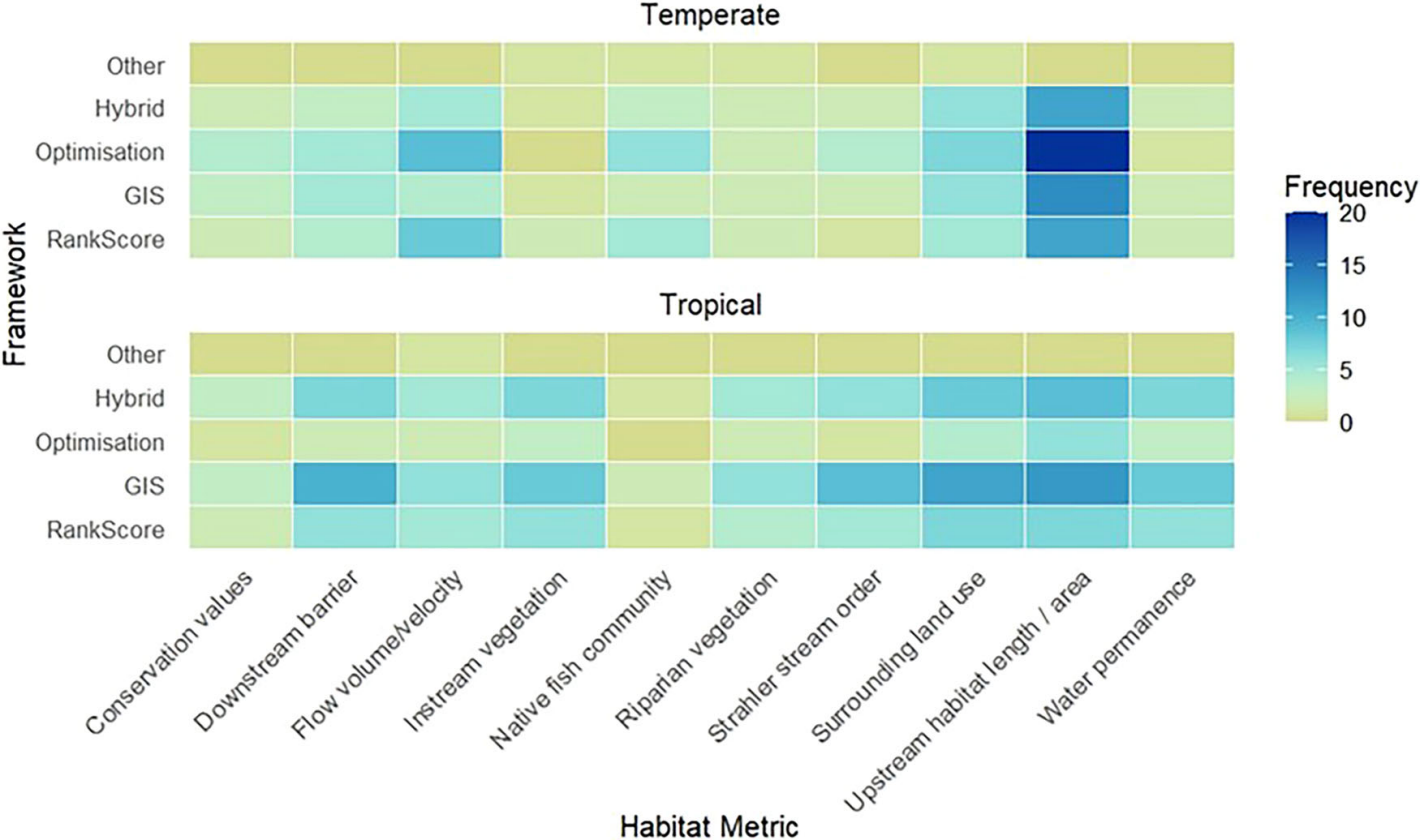
Frequency distribution of the ten most frequently used habitat metrics in all reviewed frameworks in relation to framework type and regional focus

**Fig. 7:**
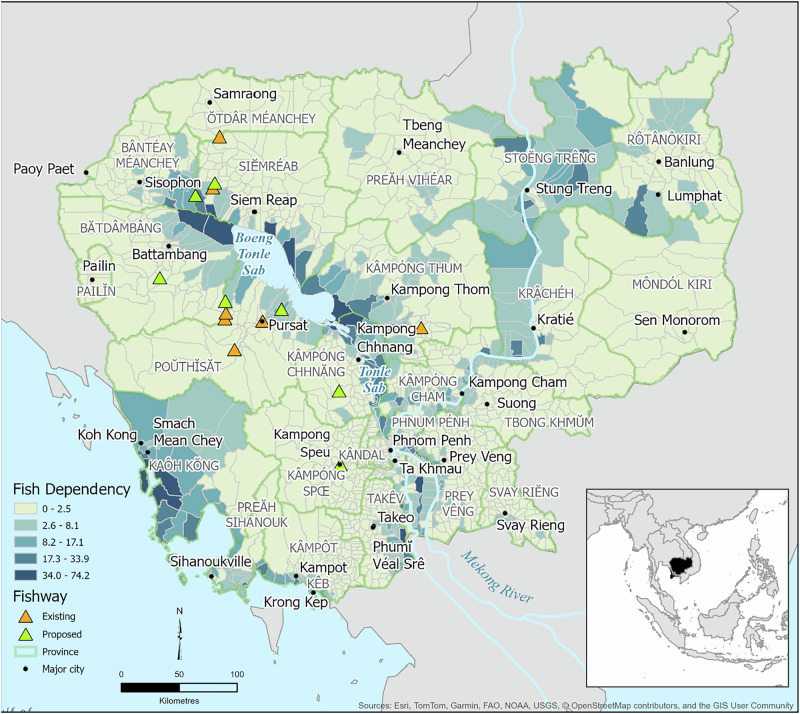
The location of fishways built (orange triangles) and planned (green triangles) in Cambodia to date show existing local community dependence on fish for nutrition or livelihoods has not influenced barrier prioritization. For example, fishways are absent from Tonlé Sap Lake’s northern tributaries where communities are highly dependent on fisheries (Save Cambodia’s Wildlife 2015). While this map highlights areas of existing need, this approach can also be used to anticipate and prioritize areas with emerging or future nutritional vulnerability

Framework attributes, including date, publisher, geographic focus, framework method (e.g. rank and score, GIS, optimization, hybrid), and metrics were extracted and analyzed in Microsoft Excel. The results were used to visualize the frequency distribution of prioritization frameworks published over time according to geographic area (Fig. 4), the frequency distribution of social metrics included in prioritization frameworks (Fig. 5) and the frequency distribution of the 10 most common habitat metrics observed in prioritization frameworks according to both framework method (i.e., rank and score, GIS, optimization, or hybrid) and geographic focus (Fig. 6). Academic frameworks referred to frameworks published in peer-reviewed journals whereas agency frameworks related to frameworks published by natural resource management or fishway implementation agencies, often identified from gray literature. To observe the spatial relationship between fishway locations and fishery dependency, spatial data published by Save Cambodia’s Wildlife (2015) on Open Development Cambodia were overlaid with the author’s spatial dataset of existing and planned fishways in Cambodia (Fig. 7).

## Results

### General Trends in Fish Passage Barrier Prioritization Frameworks

Of the 93 publications analyzed, most frameworks (*n* = 49, 52%) focused on restoring fish passage in North America, followed by Europe (19, 18%), Oceania, (15, 16%), southeast Asia (7, 8%), South America (2, 2%), and Africa (1, 1%) (Fig. 4). Twenty-one of 93 frameworks reviewed (23%) focused on fisheries in tropical latitudes of which 11 (52%) were developed for Queensland, Australia, 7 (33%) for LMB, southeast Asia, and 1 (5%) each for Florida, North America; Brazil, South America; and Africa. The LMB was the best represented of regions where fisheries are a critical food resource. Analysis of reviewed framework publication dates showed an increasing trend until 2020 when publication frequency sharply declined (Fig. 4).

### Social and Nutrition Metrics Rarely Occurred

Overall, social metrics including nutrition security were under-represented in reviewed frameworks (Fig. 5) likely due to the fish biology focus of most practitioners. Although we found seven publications presenting prioritization frameworks for the LMB where fish are integral to nutrition security, these referred to only three unique frameworks (Cooper et al. 2019; Marsden et al. 2023; Ziv et al. 2012), whereas Baumgartner et al. (2016), Horta et al. (2023), Marsden et al. (2014), and Mekong River Commission (MRC 2023) presented iterations of a framework ultimately published under one of our authors’ names in Marsden et al. (2023). Of the three unique frameworks, only one included nutrition criteria (Cooper et al. 2019). Cooper et al. (2019) presented a cost-benefit analysis tool assessing individual barriers including assessment of estimated nutritional benefit of improved fish passage to upstream communities (Cooper et al. 2019). Food and nutrition security were noted as motivating fishway implementation in the introductory sections, but not in the framework metrics, of five frameworks, all of which focused on the LMB (Horta et al. 2023; Marsden et al. 2014; 2023; MRC 2023; Ziv et al. 2012). Nutrition security metrics were not directly integrated into Marsden et al. (2023) which was the only framework aimed at systematic basin-wide (as opposed to individual) barrier prioritization for fish passage in the LMB. Ziv et al.’s (2012) framework, which focused on hydropower effects on migrating fisheries likewise did not integrate nutrition into its criteria. Nutrition security metrics were not observed in frameworks reviewed beyond the LMB region except for one Queensland, Australia focused framework (Carter et al. 2007), which included provision for a metric concerning the nutritional importance of a fishery impacted by barriers, (designed to be calibrated by local indigenous groups), but did not actually calibrate or test it.

### Ecological and Habitat Metrics Occurred Frequently, Especially Longitudinal Habitat Connectivity Metrics

Reviewed framework criteria more frequently considered large riverine and hydropower dams, culverts, and smaller weirs (83%) than floodplain irrigation infrastructure like canals, levees, and sluice gates (17%). Of the 16 frameworks that explicitly referred to irrigation, most (62.5%) related to tropical contexts. Ecological habitat focused metrics were also common (Fig. 6). Upstream habitat length/area was the most common habitat metric in both tropical and temperate focused frameworks whereas lateral habitats rarely featured (9%). In temperate regions, optimization frameworks were more likely to use upstream habitat length/area, whereas this metric was more common in GIS based frameworks in tropical regions. Instream vegetation metrics contributed more to tropical frameworks, possibly given the lowland geography of the tropical river systems they considered (e.g., Queensland Australia east of the Great Dividing Range and LMB) as opposed to the steeper, faster flowing, less vegetated salmonid habitats considered in temperate north American contexts. Likewise, more is known about target fish (e.g., salmonid) population status in temperate regions allowing native fish demographic and community data to inform framework metrics there, whereas in immensely biodiverse tropical regions, conservation focus is commonly diffused across multiple, often data deficient species (e.g., in the LMB).

## Discussion

Many barrier prioritization frameworks exist; however, human nutrition security is rarely integrated into decision-making metrics. Overall, there was little evidence that human nutrition tangibly influenced fish passage prioritization in any of the frameworks reviewed. This is particularly concerning in the LMB context where nutrition security objectives now underpin fishway investments. The exception was Cooper et al. (2019), an LMB focused framework, which included an estimate of the nutritional advantage of improved fish passage but fell short of basin-wide assessment. In contrast, Marsden et al. (2023), which was also written for the LMB, offered a basin wide barrier assessment tool which acknowledged maintaining fisheries as food as a motivation for fish passage investment, but did not directly include human nutrition security prioritization criteria.

### Current Frameworks Lack Barrier Prioritization Metrics to Support Fish Passage Restoration for Human Nutrition Security Objectives

Serious challenges remain in operationalizing the intention to maintain inland capture fisheries for the nutrition security of dependent human populations in the LMB and beyond (Béné et al. 2016; Kandulu and Connor 2017). Overall, frameworks maintain a technical and ecological focus, yet rivers are used and perceived differently across regions (Anderson et al. 2019). In the LMB rivers are vital food sources for much of society, while in North America, they are framed more through environmental science and conservation. Frameworks developed in North America and temperate regions are ecologically unsuited to tropical contexts but also appear incognizant of benefits people gain from wild fish as a food resource. Uncritically applied, such frameworks may be severely hampered by data paucity for highly diverse tropical fisheries and by overlooking the question of how fish passage technology can support nutrition security objectives. We theorize this reflects the emergent trajectory of fish passage technology development from roots in temperate contexts as a technical (engineering) solution to refinements incorporating local fish ecology (Twardek et al. 2022), and which now, in deployment in new contexts, needs to integrate the social need and benefits of fisheries to people, including as food. Although fish are often seen as more important for food in tropical regions, the nutritional value of inland recreational fisheries is also becoming increasingly clear in temperate areas where recreational fishing is typically considered non-consumptive (Embke et al. 2022; Lynch et al. 2024). For example, Embke et al. (2020) showed that intense fishing in Lake Wisconsin led to local fish consumption nearing the U.S. average. Yet, the nutritional value of recreational fisheries and the long recognized cultural and dietary importance of inland fisheries for First Nations in North America, Australia, and New Zealand remain largely absent from mainstream fisheries management (Atlas et al. 2021; Marushka et al. 2021; Noble et al. 2016).

### Current Barrier Prioritization Frameworks should be Extended to Include Metrics Which Link Fish Passage Restoration to Human Nutrition Security Outcomes

Globally, the evolution of fish passage barrier prioritization frameworks can benefit from clarifying and integrating nutritional advantages of improving fish passage. This work will require the integration of a broader range of experts into fish passage design teams (Wainger et al. 2023), which has already demonstrated benefits in the LMB (e.g., Baumgartner et al. 2016) and abroad. For example, in the Columbia River Basin, multi-partner projects have been more likely to make progress towards salmon recovery (Hill and Kolmes 2024). Collaboration of local district, provincial, or national health departments, citizen scientists, local markets, or demographic statisticians for example, with fisheries scientists could enrich barrier prioritization (Table 2, Melesse et al. 2020; NOAA 2021). Multi-disciplinary collaboration can broaden the range of metrics included in barrier prioritization to include macro and microscale nutrition-sensitive metrics related to food productivity, output, and consumption, and which link to various multilateral environmental agreement mechanisms (Lynch et al. 2025) and SDGs (Melesse et al. 2020). For example, health statistics reported by district and provincial hospitals generate information about the spatial distribution of nutritional needs which can be overlayed with existing systematic basin wide barrier assessments. The use of human data makes human ethics in research and data use compliance essential. Collaboration can also assist with fish passage implementation as social-political factors often shape how local communities benefit from increased fish supply (Baird et al. 2020; Friend et al. 2012; Nguyen-Khoa et al. 2020). We provide a simplified example focusing on Cambodia in the LMB which has amongst the highest fish consumption rates per capita globally (Sean and Sithirith 2024, Fig. 7). In Fig. 7, we leveraged the most recent publicly available dataset about fish dependency, published by a Cambodian non-governmental organization (NGO), and overlaid it with the point locations of existing and planned fishways in Cambodia. Nasielski et al. (2013) define fish dependency by economic type indicators such as number of people engaged in fishing and boats per household. These indicators however likely correlate to nutritional dependence given an average 43% of catch is kept for home consumption, with fish assuming increased importance to the wellbeing of disadvantaged Cambodian households (Mousset et al. 2016). Figure 7 demonstrates the lack of spatial relationship between dependence on fisheries and fishway location and illustrates how integrating collaborative data (Table 2) can highlight where barrier remediation might contribute most to the nutrition and livelihoods of local communities. We do not suggest that present fishway locations are unjustified. For example, our study highlighted various technical and ecological metrics which contribute to barrier prioritization. Rather, we acknowledge nutritional need as one of multiple considerations involved in barrier prioritization.

Nutrition sensitive metrics could be integrated into barrier prioritization directly or indirectly. Directly triangulating fishway trapping surveys with published fish nutritional composition data (e.g., Barrett 2020; Nam and Bunthang 2011; Thilsted 2012) is one option. Cooper et al.’s (2019) method for example could be expanded to include micronutrients (e.g., iron, calcium, omega-3s) needed by SDG 2 focal demographics like women and children (Ritchie and Roser 2017). Involvement of local communities in household consumption surveys, complemented with fish mark and recapture surveys (Cooke et al. 2016; Gerking 1950), could determine that fish trapped and marked in fishways end up on household plates. Alternatively, integrating secondary data from fisheries or relevant agencies may promote multi-partner collaboration and provide a useful, broader view (Table 2 - Lynch et al. 2020; FAO et al. 2022).

An indirect option is to critically evaluate which habitats are most relevant to local nutrition security and ensure these are represented in fish passage barrier prioritization. Habitat type has implications for who benefits from improved fish passage (Arthur and Friend 2011; Conallin et al. 2019) and speaks to SDG 5: Gender equality. For example, in Cambodia where women and children are most affected by nutrition insecurity, specifically targeting the habitats they fish may improve demonstrable outcomes for those groups. This may mean greater representation of streams or floodplain wetlands including rice fields harvested by family fishers, women, and children (Garaway 2005; Garaway et al. 2013; Freed et al. 2020).

### Barrier Prioritization Frameworks can be Evaluated after Use to Determine If They are Relevant and Impactful

**Fig. 8:**
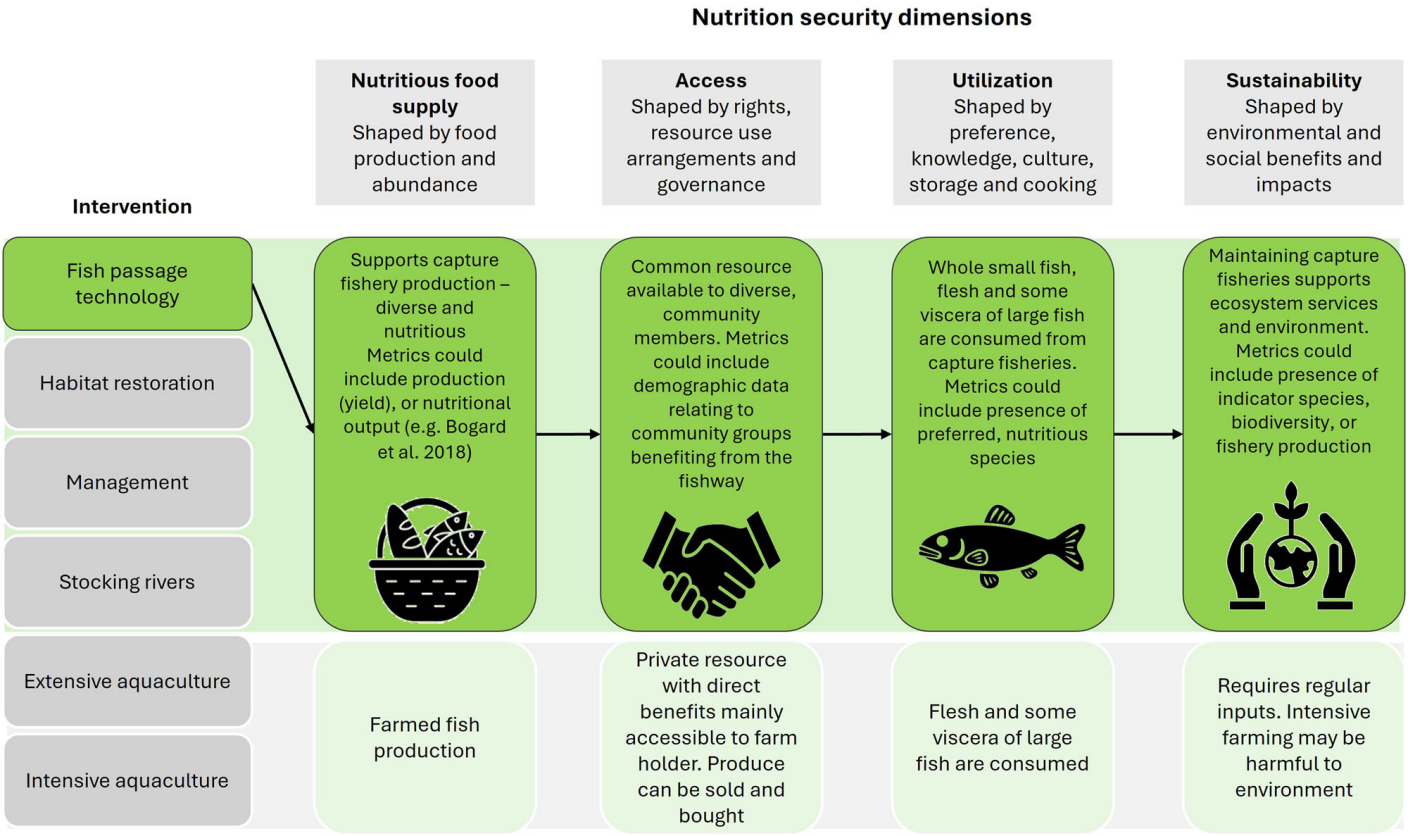
Fish passage is a foundational intervention that supports other fishery restoration efforts aimed at maintaining capture fishery populations and the benefits they provide to ecosystems and human communities. Explicitly, effective fish passage restoration supports production of free, indigenous, accessible, nutritious food-fish as a shared resource. Here, the four pillars of nutrition security (Simelane and Worth 2020) frame how nutrition-sensitive metrics could potentially be integrated into fish passage barrier prioritization frameworks. However, fishway benefit realization is contextual, highlighting the importance of collaboration with in-country partners to enable long-term benefits, especially for vulnerable community members

Following prioritizing barriers to support human nutrition security, the next logical step is evaluating the effects of barrier remediation on local community health outcomes. Evaluation is a step often missed in fish passage project implementation but is essential to determining the effectiveness of not only fishways themselves but also of the prioritization process (Duncan et al. 2024). Prioritization frameworks, like other fisheries interventions are likely more effective when tailored to their specific application context (de Silva et al. 2017; Nguyen-Khoa et al. 2020). That is, the suitability of a given framework depends on how well it matches the place, intended users, and social-ecological conditions (de Silva et al. 2017). Criteria can include the aims of prioritization (e.g., support human nutrition security) and fish passage principles (e.g., ecology, technology). Aligning framework criteria to local contexts can help strengthen confidence for investors that restoration action is meaningfully contributing to social well-being. The metrics used to integrate nutrition security into fishway prioritization frameworks need not detract from framework utility or accessibility to diverse, local users (Conallin et al. 2022; Duncan et al. 2024; Nieuwlaat et al. 2021). The issue is not about choosing between ecology and human health but rather to ensure important decisions about fisheries investments are based on scientific and socially defensible criteria. Moreover, fishway implementation occurs within complex social, technical, and ecological contexts that shape the ability of implementers and their partners to realize their benefits (Nguyen-Khoa et al. 2020). Likewise, the benefits of fishways, which contribute to maintaining or increasing the supply of nutritious food and in turn enhance opportunities for supporting human health, economic livelihoods, and community empowerment (in the framing of nutrition sensitive agricultural systems – refer to Ruel et al. 2018) may be amplified when complemented by governance and management interventions like fishing, water, and riparian land use regulations, and household nutrition training (Fig. 8, Nguyen-Khoa et al. 2020). The intersections between these interventions further underscore the importance of cross-sector collaboration to leverage expertise, define meaningful metrics, and deliver both fishway and Sustainable Development Goals (Barbour et al. 2016).

In many tropical contexts, integrating human nutrition security criteria into barrier prioritization is a logical step in steering fish passage investments towards achieving SDG 2 and related goals like SDG 1: No poverty, SDG 3: Good health and wellbeing, and SDG 5: Gender equality through explicit consideration of fishway beneficiaries; as well as SDG 6: Clean water and sanitation for all and SDG 15: Life on land which refer to the ecological integrity of inland aquatic food production systems (Lynch et al. 2017; 2020). Establishing human health and/or livelihoods metrics pre- and post-fishway implementation can allow evaluation of nutritional effects and improve the probability of fishways delivering on nutrition security objectives. A useful case study may be Cambodia’s Tonlé Sap region where human nutrition security is intricately linked to capture fisheries (Barrett 2020; Hortle et al. 2008). More broadly the present work has relevance given the importance of inland fisheries to nutrition security for diverse human communities worldwide (Lynch et al. 2020). Although human health and fishery monitoring can provide an indication of fishway effectiveness, it is important to acknowledge fishways represent one of many fishery restoration interventions, and the role of other factors, including declining stream flow, pollution, invasive species, improved management regimes, and climate change which also influence aquatic ecosystem and human health.

## Conclusion

The UN SDGs and other multilateral environmental agreements (e.g., Convention on Biological Diversity) implore scientists and managers to consider social and ecological outcomes in landscape and water resource development, including by ensuring adequate amounts of nutritious food are reliably available to vulnerable people. Fish passage prioritization frameworks provide an entry point for integrating SDGs and other global targets into fishway implementation, and fishway implementers can develop decision-making tools that are iterative yet adaptable to the local context.

Our critical examination of prioritization frameworks highlighted differences among fishway implementation contexts and showed that people use rivers differently. If a user depends on the river for nutrition security, integrating prioritization framework criteria that reflect that need may improve achievement of that goal. Agencies are investing in fish passage technology to support nutrition security, but present decision-making frameworks lack relevant metrics, so are they achieving value for money? We suggest that where nutrition security is relevant to fishway performance, the prioritization frameworks guiding fishway implementation may need fundamental reconsideration to ensure fish passage investments (likely totaling hundreds of millions of dollars per year globally) have the best chance of supporting nutrition security goals like SDG 2. Better integration of fish ecology and human health data into the prioritization process is essential and further research could help to evaluate the benefits provided by fish migrating through fishways. On-ground application and continued refinement of these concepts could help ensure these frameworks not only improve fish passage infrastructure but also help support better nutrition and food production for the communities who need it most.

Any use of trade, firm, or product names is for descriptive purposes only and does not imply endorsement by the U.S. Government.

## Data Availability

No datasets were generated or analyzed during the current study.
